# A Retrospective Cohort Analysis of Transarterial Chemoembolization for Hepatocellular Cancer at a Tertiary Center in Switzerland

**DOI:** 10.3390/jcm13113279

**Published:** 2024-06-02

**Authors:** Fabian Haak, Tobias Karli, Martin Takes, Christoph J. Zech, Otto Kollmar, Savas D. Soysal

**Affiliations:** 1 Clarunis, Department of Visceral Surgery, University Digestive Health Care Center, St. Clara Hospital and University Hospital Basel, 4058 Basel, Switzerland; 2 Department of Visceral, Transplant, Thoracic and Vascular Surgery, Division of Hepatobiliary Surgery and Visceral Transplant Surgery, University Hospital Leipzig, 04103 Leipzig, Germany; 3 Interventional Radiology, Radiology and Nuclear Medicine, University Hospital Basel, University of Basel, 4001 Basel, Switzerland; 4 Medical Faculty, University of Basel, 4001 Basel, Switzerland

**Keywords:** hepatocellular carcinoma, oncology, interventional therapy

## Abstract

**Background/Objectives**: International guidelines recommend transarterial chemoembolization (TACE) for intermediate-stage hepatocellular carcinoma (HCC). However, it is used outside these recommendations and has proven beneficial in prolonging survival. Since the role of TACE outside BCLC stage B is unclear, the present study analyzed the results of TACE performed at a tertiary center in Switzerland for different treatment groups, and aims to highlight the treatment outcomes for these groups. **Methods**: This retrospective cohort study includes 101 HCC patients undergoing TACE at our center. Patients were further subdivided into groups according to therapy combinations (therapies applied before and after index TACE). Kaplan–Meier survival curves were calculated for the Barcelona Center for Liver Cancer (BCLC) subgroups. **Results**: After TACE, the median survival was 28.1 months for BCLC 0, 31.5 months for BCLC A, 20.5 months for BCLC B, 10.8 for BCLC C, and 7.5 months for BCLC D. A lesion size larger than 55 mm was negatively associated with survival (HR 2.8, 95% CI 1.15–6.78). Complications occurred after TACE procedures: Clavien–Dindo I + II = 30, Clavien–Dindo > 3 = 2. **Conclusions**: TACE was performed in a substantial part of our cohort outside of routinely used treatment guidelines. The combination of the survival data and complication rate in these patients suggests it was a safe and beneficial strategy. Furthermore, our data show that in our cohort, the survival benefit associated with TACE was restricted to patients with a lesion size smaller than 55 mm.

## 1. Introduction

Primary liver cancer represents a global healthcare problem. It has the 6th highest incidence and the 3rd highest mortality of all cancers worldwide. Globally, for males, it has the 5th highest incidence and the 2nd highest mortality, and for females, the 9th highest incidence and 6th highest mortality [1,2,3,4]. Hepatocellular carcinoma (HCC) is the most common form of primary liver cancer, accounting for three-quarters of all liver cancers [5,6]. The incidence varies substantially throughout the world, being higher in Asia and Africa, and lower in Western Europe and North America [7]. Also, across Europe, differences in incidence can be seen, with more cases in southern Europe compared to the northern countries. The main risk factors for the development of HCC are chronic viral hepatitis and alcohol abuse, leading to liver cirrhosis. The Barcelona Clinic Liver Cancer (BCLC) staging system is the most commonly used system to classify HCCs and has recently been revised [8,9,10,11,12]. Sadly, most HCCs discovered are not amenable to curative treatment, either because of delayed diagnosis or because of complicated end-stage liver disease [13,14]. Therefore, non-invasive treatment options have been used to treat patients in the non-curative stages. Studies have shown that transarterial chemoembolization (TACE) can improve intermediate-stage survivals [15,16]. Therefore, the BCLC staging system recommends TACE for intermediate-stage HCC (BCLC B) [8,9,10,17]. However, at most liver centers, including our hospital, other BCLC stages than BCLC B are also treated occasionally by TACE [18]. Despite the clear guidelines for BCLC B, the role of TACE in other stages remains less defined, necessitating a detailed analysis of its efficacy across a broader spectrum of clinical scenarios. This study aims to bridge this knowledge gap by providing comprehensive data on the outcomes of TACE in various BCLC stages at a tertiary center in Switzerland. By integrating literature on various treatment options—inside and outside the current guidelines—with local clinical data, we seek to enhance the existing literature and, thereby, provide further insight into the broader applicability and efficacy of TACE, thus potentially influencing future treatment protocols and guidelines.

## 2. Materials and Methods

This is a retrospective cohort study analyzing the survival of 101 patients who received TACE at our tertiary hospital in Switzerland between 2010 and 2020. All patients who received TACE treatment at our hospital during this period were included in the study. Twenty-seven patients were excluded if they only visited our center for the TACE intervention without further follow-up. The ethics committee of Northwest Switzerland approved the study (registration number 2020-00076). HCC was diagnosed following current guidelines [17,19]. Patients were either primarily seen at the tertiary center or referred from two other tertiary centers in the area that also treat HCC but do not perform TACE. Data were collected retrospectively via patient chart analysis by an independent member of our research team trained for this purpose and reviewed by the same team. Additional information was gathered by calculation (Child–Pugh, American Association of Anaesthesiologists (ASA) score, Model for End-Stage Liver Disease (Meld) Score, Aspartate Aminotransferase to Platelet Ratio Index (APRI), and Charlson Comorbidity Index (CCI)) at the time of intervention. To report radiological signs of cirrhosis, the following indicators were used: nodular liver, flattening of the left edge, biconvex organ shape, increased echogenicity with irregular areas, enlarged liver, rarified venous system of the liver, ascites, splenomegaly, portal vein thrombosis, recanalization, hepatofugal paraumbilical venous flow, and irregular liver surface. A multidisciplinary team managed the patients, and treatment decisions (e.g., performing TACE) were made at multidisciplinary conferences within and outside the BCLC treatment algorithm. TACE was performed by using doxorubicin-loaded beads exclusively (from 2010 to 2014 with 100–300 micron DC Beads, (Merit Medical, South Jordan, UT, USA) from 2014 until recent with 100 micron Tandem Beads (Boston Scientific, Marlborough, MA, USA). The procedures themselves were performed per current internal and external guidelines and were thus comparable. The main varying factor was the extent of treatment, corresponding to the individuals’ tumor burden. The endpoint was survival measured from the date of the first TACE performed until death. Kaplan–Meier and Cox proportional hazard models were performed to check survival data. The Kruskal–Wallis test was performed to look for significant survival differences between BCLC subgroups. Specific differences in significance for the separate subgroups were determined by performing pairwise Mann–Whitney tests with Bonferroni correction. A *p*-value under 0.05 was considered statistically significant. The data analysis was performed using Stata15 (StataCorp LP, College Station, TX, USA) and R studio (v4.2.3, R Core Team, 2023).

## 3. Results

In total, 101 patients with 186 interventions were included in the analysis. The mean age of the patients was 67.5 ± 10.1 years. 24.8% were female (25 patients), and 75.2% were male (76 patients). The mean BMI of the patients was 27.7 ± 5.9. The mean CCI was 8.5 ± 3. The average applied dosage of Doxorubicin was 96.7 mg (SD ± 37.0 mg) (see Table 1 for patient characteristics).

Of the treated patients, 84.2% (*n* = 85) had histologically confirmed liver cirrhosis. Of these 85 patients, 65.9% (*n* = 56) had a hepatic venous pressure gradient (HVPG) > 10 mmHg, and thereby, by definition, a clinically relevant portal hypertension. Of the patients treated, 15.8% (*n* = 16) did not have histologically confirmed cirrhosis. Of these sixteen patients, three patients showed two or more radiographic signs of liver cirrhosis, and six showed one radiographic sign of liver cirrhosis. The evaluation of further characteristic scores showed a mean MELD score of 10.4 ± 4.4 and a mean APRI score of 2.4 ± 4.7. Fifty patients (49.5%) were classified as Child–Pugh Class A, 45 patients (44.6%) as Class B, and 6 patients (5.9%) as Class C. Analysis concerning the correlation of clinical scores used to assess the patient’s liver health was performed. Meld vs. Child showed a rho value of 0.53 (*p* < 0.001), Meld vs. APRI score showed a rho value of 0.28 (*p* = 0.005), and Child vs. APRI score showed a rho value of 0.26 (*p* = 0.01). Thus, there was a moderate positive correlation between MELD and Child-Pugh scores, a weak positive correlation between MELD and APRI scores, and a weak positive correlation between Child–Pugh and APRI scores.

The hepatoma arterial embolization prognostic (HAP) score was calculated for our cohort [20]. The score predicts outcomes in patients with HCC undergoing TACE. It can further be used to predict outcomes during repeated TACE [21]. Patients with A–B-class risk scores show significantly better survival than those with C–D-class risk scores. In our cohort, no patients had an A-class risk score. Sixteen patients had a B-class risk score, 49 patients had a C-class risk score, and 10 had a D-class risk score. Our cohort showed a significant correlation between HAP score and survival with a hazard ratio of 1.82 (*p* = 0.006, 95% CI 1.19–1.78). This is in line with previously published data [20,21]. 

The distribution of BCLC stages of the patients who received a TACE was as follows: BCLC 0 = 4 patients (4%), BCLC A = 31 patients (30.7%), BCLC B = 50 patients (49.5%), BCLC C = 10 patients (9.9%) and BCLC D = 6 (5.9%).

Several other therapies to treat HCC exist which can be combined with TACE. The most common treatment combinations in our cohort were as follows: 23 patients received a single TACE, 16 patients received two TACE interventions, 8 patients received a TACE followed by surgery, 8 patients received three TACE interventions, 5 patients received four TACE interventions, and 4 patients received more than four TACE interventions. For an overview of distribution, please see Table 2 for the characteristics of the different subgroups.

Complications occurred during or after 32 of 186 interventions (17.2%). Most of these complications were Clavien–Dindo grade 1 (*n* = 12) and grade 2 (*n* = 18). There were two Clavien–Dindo grade 5 complications (Sepsis with ensuing death). No Clavien–Dindo grade 3 or 4 could be observed (Table 3).

The median survival of all treated patients was 21.4 months (IQR 11.7–37.6). The probability of survival of the entire cohort at 1, 2, 3, 4, and 5 years were 72.3%, 36.6%, 25.7%, 16.8%, and 8.9% respectively. For the subgroup of patients with BCLC ≤ A4 and B, the probability of survival at 1, 2, 3, 4, and 5 years were 80%, 41.2%, 29.4%, 20%, and 10.6%. The median survival of all subgroups according to the BCLC stage was as follows: BCLC 0 = 28.1 months (IQR 22.8–35.7), BCLC A = 31.5 months (IQR 18.1–61.7), BCLC B = 20.5 months (IQR 12.6–29.4), BCLC C = 10.8 months (IQR 3.9–16.3), and BCLC D = 7.5 (IQR 2–11.6) (Table 4).

The mean survival of the respective groups is listed in Table 4. Please see Supplementary Materials Figures S1 and S2 for box plots and scatter plots. A Kruskal–Wallis rank-sum test showed a significant difference in survival comparing the BCLC subgroups (*p* < 0.001). Pairwise Mann–Whitney tests with Bonferroni correction showed a significant difference in survival between specified BCLC groups (Supplementary Materials Table S1).

Differences in survival between all the BCLC groups.

**Figure 1:**
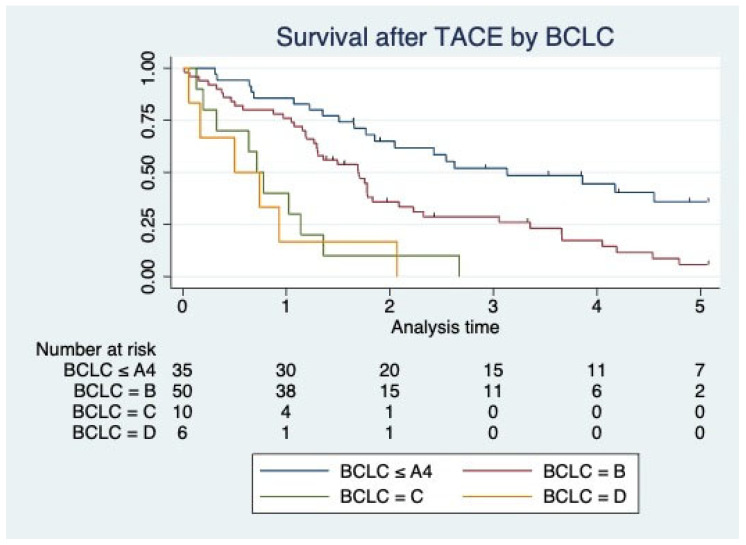
Kaplan–Meier curves for survival by the BCLC subgroup.

Survival according to the BCLC stage is additionally depicted by Kaplan–Meier curves (Figure 1). 

Univariate analysis showed that lesion diameters up to a size of 55 mm did not significantly impact survival. This changed for lesions of 55 mm or more in size, which were associated with a worse survival (hazard ratio 2.8 (95% CI 1.15–6.78)). Further analysis showed a significant negative impact of bilobular disease (HR 2.11, CI 1.31–3.39), CCI (HR 1.08, CI 1.01–1.17), higher Child score (HR 1.37, CI 1.18–1.60), higher MELD score (HR 1.06, CI 1.01–1.12), M1 vs. M0 (HR 3.14, CI 1.58–6.23), and higher number of lesions (HR 1.19, CI 1.02–1.40) on survival (Table 4).

## 4. Discussion

This retrospective single-center analysis of a TACE cohort from Switzerland illustrates the survival of HCC patients treated within or outside current treatment guidelines. It also shows factors associated with a better survival, such as a lesion size under 55 mm, possibly implicating tumor features, which should be considered before choosing TACE as a treatment.

Previously published literature shows survival after TACE at one year ranging between 13 and 82%, and two years running between 11 and 63% [15,22]. The lower survival rates come from studies with low patient numbers. One specific prospective trial, including patients with only BCLC ≤ A4 and BCLC B, reported survival at 1, 2, and 3 years at 82%, 63%, and 29% for chemoembolization, respectively [22]. When comparing this to our results, we can see that the same subgroups achieve similar survival rates, except for survival at year two, which is lower in our cohort (41.2% vs. 63%). 

This survival compares to other modalities. Radiofrequency ablation (RFA) has been reported to have better survival. Liu et al. report survival for 1, 3, and 5 years at 97%, 83%, and 66%, respectively [23]. Wong et al. report a slightly worse survival rate of 91.6% (1 year), 73.5% (3 years), and 57.4% (5 years) [24]. Finally, Gory et al. report survival at 62% (3 years) and 37% (5 years) for lesions smaller than 5 cm [25]. RFA is a treatment form with curative intent. This sets it apart from TACE and can explain the better survival of the above-mentioned studies as patients with earlier disease stages are selected for treatment with a subsequently better survival. Data for RFA-treated patients with more advanced stages are hard to come by as this treatment form is not recommended for this patient group. A study analyzing the survival of a combined treatment with tremelimumab and RFA states a median overall survival of 12.3 months [26]. This is lower compared to our reported median survival of 21.4 months. Microwave ablation (MWA) is another possible therapy modality. Swan et al. report a one-year survival of 72.8% [27], which is comparable to our reported survival at one year. Ohmoto et al. reported a higher survival after MWA, with 89% (1 year) and 49% (3 years) [28]. Here, the patient cohort was confined to small HCCs, explaining the higher survival observed.

The HAP score was developed to predict outcomes in patients with HCC undergoing TACE. Our analysis also showed that a higher score was associated with a worse survival (HR 1.82, *p* = 0.006), in accordance with the published literature [20]. 

TACE is recommended for intermediate-stage HCC (BCLC B) [17]. However, studies suggest that it can also improve survival in higher stages (e.g., BCLC C) [29,30,31,32,33,34]. TACE was beneficial in patients with extrahepatic spread, irrespective of Sorafenib [35]. Chung et al. reported a median survival of 7.4 months (range 1.7–33.3) for patients with main portal vein invasion who received a TACE compared to 2.6 months when only receiving the best supportive care [36]. Other groups demonstrate the superior outcome of TACE + Sorafenib over TACE alone [37,38]. The overall survival was reported to be 8.9 months in the combined group. Our BCLC C cohort showed a better median survival of 10.8 months (IQR 3.9–16.3). In our BCLC C and D cohort, two patients received sorafenib combined with TACE. The results of the above-mentioned studies emphasize the importance of the achieved survival at our center. Only two of the ten patients in the BCLC C group received repeated TACE, showing that this fragile group can achieve a positive effect with limited invasiveness. In the literature, there are also data demonstrating a better survival: Hirst et al. reported a median survival of 21 months for BCLC C patients [39]. A group from Brazil showed a median survival of 12 months for advanced-stage HCC patients [40]. 

Repeated TACE is recommended as long as a complete or partial response is seen (defined by mRECIST) [17,41]. However, the role of repeated TACE outside the primary recommendation for BCLC B is unclear. Parallel to the above-mentioned beneficial implications of TACE for higher BCLC stages, additional studies have also reported advantages for repeated TACE in these patients [34]. In this context, the ABCR score was developed to determine whether a repeated TACE is beneficial [42]. A score ≥ four before the second TACE identifies patients with a dismal prognosis. A calculation of the ABCR for our patients who received a Re-TACE showed a negative correlation between a higher ABCR score and survival (HR 1.305, *p* = 0.03). Our Re-TACE cohort had a mean ABCR score of 1.96 (SD 0.682). There was only one patient with an ABCR score of 4. This shows that our group of patients who received an additional TACE procedure showed characteristics of patients for which additional interventions have been shown to be beneficial for survival without increasing morbidity and mortality. This demonstrates the necessity for careful patient selection and the readiness to recommend re-TACE when appropriate. 

Two major complications (Clavien–Dindo grade 5) occurred in our TACE cohort in 186 performed interventions. This equals to a grade 5 complication rate of 1.1% and is in concordance with the rates of high-grade complications published in the literature [43,44]. The two patients died due to multi-organ failure following septicemia. This type of complication has been previously reported [45,46]. 

Lesion size is an important influencing factor of TACE efficacy. Further studies have shown that an increase in tumor diameter is significantly associated with a poorer survival [47]. On the other hand, Mukund et al. recently showed that patients with extensive lesions (>5 cm) could also be successfully treated with TACE, improving their overall survival [48]. This does not contradict that increasing lesion size has an impact on the overall survival, as patient selection and specific patient characteristics confounded the excellent survival of this cohort of patients with extensive lesions. Our data show that lesion size up to 5.5 cm is not significantly associated with survival when treated with TACE in our cohort. However, a lesion size larger than 5.5 cm has a significant negative association with survival (HR 2.8, 95% CI 1.15–6.78). We believe that our data point to the possible limitations of TACE when it comes to large lesions (>55 mm) and could be an argument for alternative treatment forms (e.g., SIRT) in this subgroup of patients. 

This study has limitations. It is a retrospective study, and therefore, only data that were recorded are accessible. Due to the retrospective nature, patient groups were not randomized. Because of the data collection algorithm, there was no control group (e.g., best supportive care during the same time frame at our institution). The evaluation of tumor response was extracted from the written reports and was not standardized; it was changed from mRECIST to LIRADS during the study period. The study’s retrospective nature allowed us to employ specific codes for BCLC stage quantification at the intervention time. This may induce a particular bias as the decision to perform TACE was made during multidisciplinary tumor boards at time points several weeks before the actual intervention, potentially underscoring the patients. Loss of follow-up is an additional problem of this study and is impossible to control. 

## 5. Conclusions

The application of TACE outside of recommended BCLC stages should be further investigated and considered, as survival data and the procedure’ s safety are encouraging. The decision to perform the intervention should be tailored to the specific patient’s disease characteristics, and additional tools, such as the HAP score, should be used to aid in the decision process. Additionally, repeated interventions should also be considered outside of the recommended stage. 

## Figures and Tables

**Table 1:** Patient characteristics.

Patient Characteristic	Overall Patients (N = 101)
Age in years [mean, SD]	67.5 ± 10.1
Gender	
Female [Amount, Percent]	25 (24.75)
Male [Amount, Percent]	76 (75.25)
BMI [mean, SD]	27.7 ± 5.9
Charlson Comorbidity Index [mean, SD]	8.5 ± 3
Cirrhosis confirmed by histology	
Yes	85 (84.2)
No	16 (15.8)
Meld Score [mean, SD]	10.4 ± 4.4
APRI score [mean, SD]	2.4 ± 4.7
Child-Pugh Score	
A [Amount, Percent]	50 (49.5)
B [Amount, Percent]	45 (44.6)
C [Amount, Percent]	6 (5.9)
BCLC stage [Amount, Percent]	
Stage 0	4 (4)
Stage A	31 (30.7)
Stage B	50 (49.5)
Stage C	10 (9.9)
Stage D	6 (5.9)
Hepatoma arterial-embolization prognostic (HAP) score [Amount, Percent]	
Risk group A (Score 0)	0 (0)
Risk group B (Score 1)	16 (15.8)
Risk group C (Score 2)	49 (49)
Risk group D (Score >2)	10 (9.9)
N/A	26 (25.5)
Amount of lesions [Amount, SD]	2.8 ± 1.5
Diameter of the greatest leison [Mean in mm, SD]	41.4 ± 31.5
Bilobular lesions [Amount, Percent]	42 (41.6)
Metastasis [Amount, Percent]	
M0	90 (89.1)
M1	11 (10.9)

**Table 2:** Characteristics of the therapy combination subgroups.

Characteristic	Single TACE (*n* = 23)	2 × TACE (*n* = 16)	Surgery Followed by TACE (*n* = 8)	3 × TACE (*n* = 8)	4 × TACE (*n* = 5)	>4 × TACE (*n* = 4)
Age in years [mean, SD]	72 ± 9.3	71.8 ± 9.3	67.9 ± 4.6	66.9 ± 9.5	64.6 ± 9.3	66.8 ± 4.3
CCI [mean, SD]	10.3 ± 3.8	7.3 ± 2.1	8.1 ± 1.9	8.5 ± 1.7	7.8 ± 0.8	8.2 ± 2.2
Amount of lesions [mean, SD]	2.4 ± 1.2	2.9 ± 1.7	2.4 ± 1.7	3.6 ± 2.1	3.2 ± 0.8	4.9 ± 2.3
Diameter biggest lesion [mean, SD]	59.3 ± 40.6	52.3 ± 41.1	35.5 ± 33.8	32.4 ± 18	51.8 ± 30.4	31 ± 6.7
Bilobular lesoions [Amount, Percent]	9 (39.1)	7 (43.8)	4 (50)	3 (37.5)	3 (60)	3 (75)
BCLC Stage [Amount, Percent]						
Stage 0	0 (0)	0 (0)	1 (12.5)	0 (0)	0 (0)	0 (0)
Stage A	5 (21.73)	5 (31.25)	2 (25)	2 (25)	0 (0)	0 (0)
Stage B	9 (39.13)	11 (68.75)	3 (37.5)	6 (75)	5 (100)	3 (75)
Stage C	6 (26.08)	0 (0)	1 (12.5)	0 (0)	0 (0)	0 (0)
Stage D	3 (13.04)	0 (0)	1 (12.5)	0 (0)	0 (0)	1 (25)
Progress [Amount, Percent]						
Yes	13 (56.5)	14 (87.5)	6 (75)	8 (100)	5 (100)	3 (75)
Growth target lesion	8 (61.5)	9 (64.3)	4 (66.7)	4 (50)	2 (40)	3 (100)
New lesion	1 (7.7)	3 (21.4)	2 (33.3)	3 (37.5)	2 (40)	0 (0)
Mixed reponse (Growth + new lesion)	3 (23.1)	2 (14.3)	0 (0)	0 (0)	1 (20)	0 (0)
Metastasis	1 (7.7)	0 (0)	0 (0)	1 (12.5)	0 (0)	0 (0)
Time to progress [mean days, SD]	164.8 ± 188.3	194.1 ± 94.8	164.5 ± 46.5	327.9 ± 325.9	384.2 ± 184.6	160.3 ± 79.6
Survival [median months, IQR]	8.2 (2–20.3)	19.2 (13.8–23)	15.5 (7.5–37.2)	20.8 (14.4–35.4)	26.7 (15.7–40.2)	18.5 (13.7–23.1)

**Table 3:** Outcomes.

Outcome	Interventions (N = 186)
Progress occured [Amount, Percent]	140 (75.3)
Growth of target lesion	84 (60)
New lesion	29 (20.7)
Mixed, Growth + New Lesion	21 (15)
Metastasis	6 (4.3)
Time to progress [Mean in days, SD]	287.4 ± 354
Complications occured [Amount, Percent]	32 (17.2)
Clavien-Dindo I	12 (6.5)
Clavien-Dindo II	18 (9.7)
Clavien-Dindo III	0 (0)
Clavien-Dindo IV	0 (0)
Clavien-Dindo V	2 (1.1)

**Table 4:** Patient survival.

Survival Characteristic	Overall Patients (N = 101)
Survival, median in months (IQR)	
Entire cohort	21.4 (11.7–37.6)
BCLC ≤ A4	30.5 (19.9–56.5)
BCLC B	20.5 (12.6–29.4)
BCLC C	10.8 (3.9–16.3)
BCLC D	7.5 (2–11.2)
Survival, mean in months (SD)	
Entire cohort	22.79 (15.29)
BCLC ≤ A4	34.15 (17.53)
BCLC B	25.03 (23.48)
BCLC C	11.87 (9.32)
BCLC D	8.77 (8.58)
Survival influenced by Lesion size, HR (95% CI)	
12–25 mm	1.09 (0.45–2.61)
25–35 mm	1.66 (0.67–4.09)
35–45 mm	2.35 (0.87–6.38)
45–55 mm	1.2 (0.46–3.15)
>55 mm	2.8 (1.15–6.78)
Univariate Analysis—Correlation with survival, HR (95% CI)	
Bilobular Disease	**2.11 (1.31–3.39)**
Charlson Comorbidity Index	**1.08 (1.01–1.17)**
Child Score	**1.37 (1.18–1.60)**
Meld Score	**1.06 (1.01–1.12)**
APRI Score	1.03 (0.996–1.08)
M1 vs M0	**3.14 (1.58–6.23)**
Size of lesion	1.01 (1.00–1.02)
Amount of lesion	**1.19 (1.02–1.40)**
BMI	1.00 (0.99–1.00)
Age	1.02 (0.99–1.05)
Sex (Male vs. Female)	0.98 (0.57–1.66)

## Data Availability

Dataset available on request from the authors. The raw data supporting the conclusions of this article will be made available by the authors on request.

## Supplementary Materials

The following supporting information can be downloaded at: https://www.mdpi.com/article/10.3390/jcm13113279/s1, Figure S1. Boxplot of survival in days according to BCLC subgroup; Figure S2. Scatter plot of patient survival in days according to BCLC subgroup; Table S1. Pairwise Mann-Whitney tests.
