# Supplementary material for: A Retrospective Cohort Analysis of Transarterial Chemoembolization for Hepatocellular Cancer at a Tertiary Center in Switzerland
Source: J Clin Med. 2024 Jun 2;13(11):3279. doi: 10.3390/jcm13113279 (PMC11172573; doi:10.3390/jcm13113279)
Supplement: Supplementary file 1 [file jcm-13-03279-s001.zip › jcm-3010856-supplementary.pdf]

Journal of
Clinical Medicine

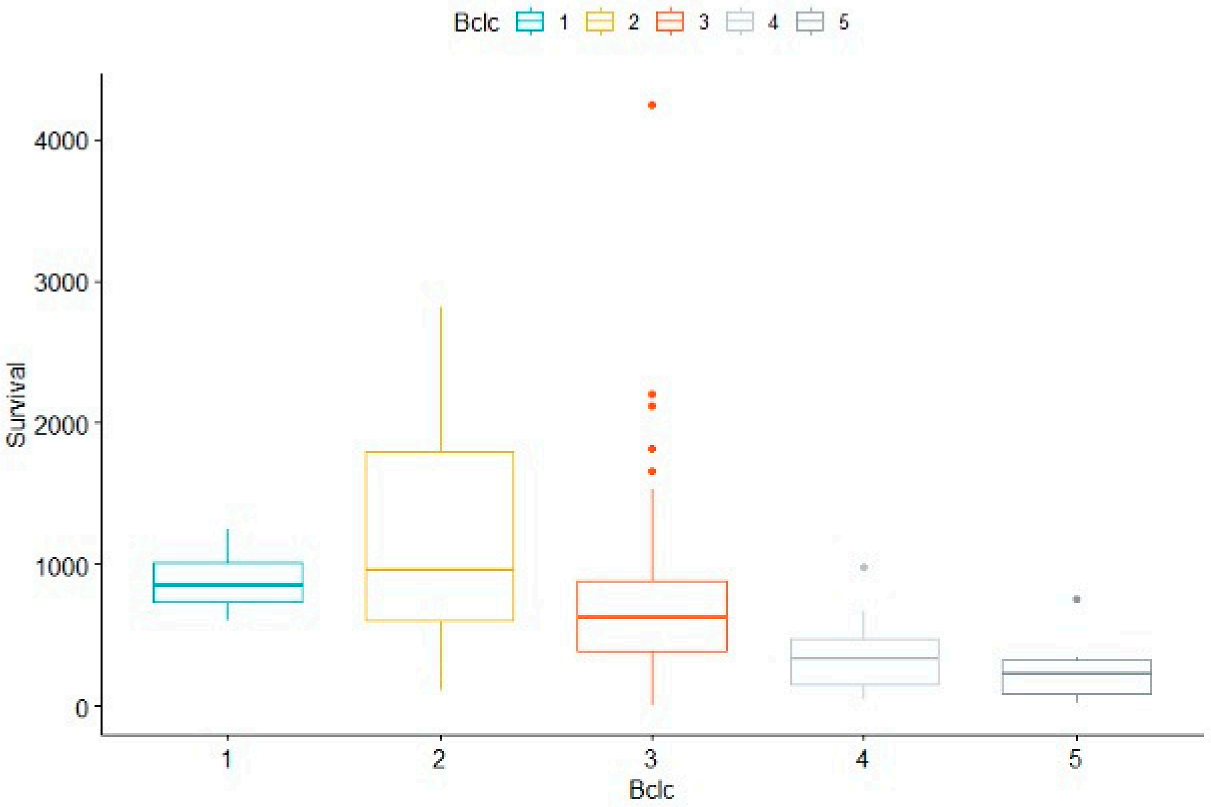

Figure S1. Boxplot of survival in days according to BCLC subgroup.

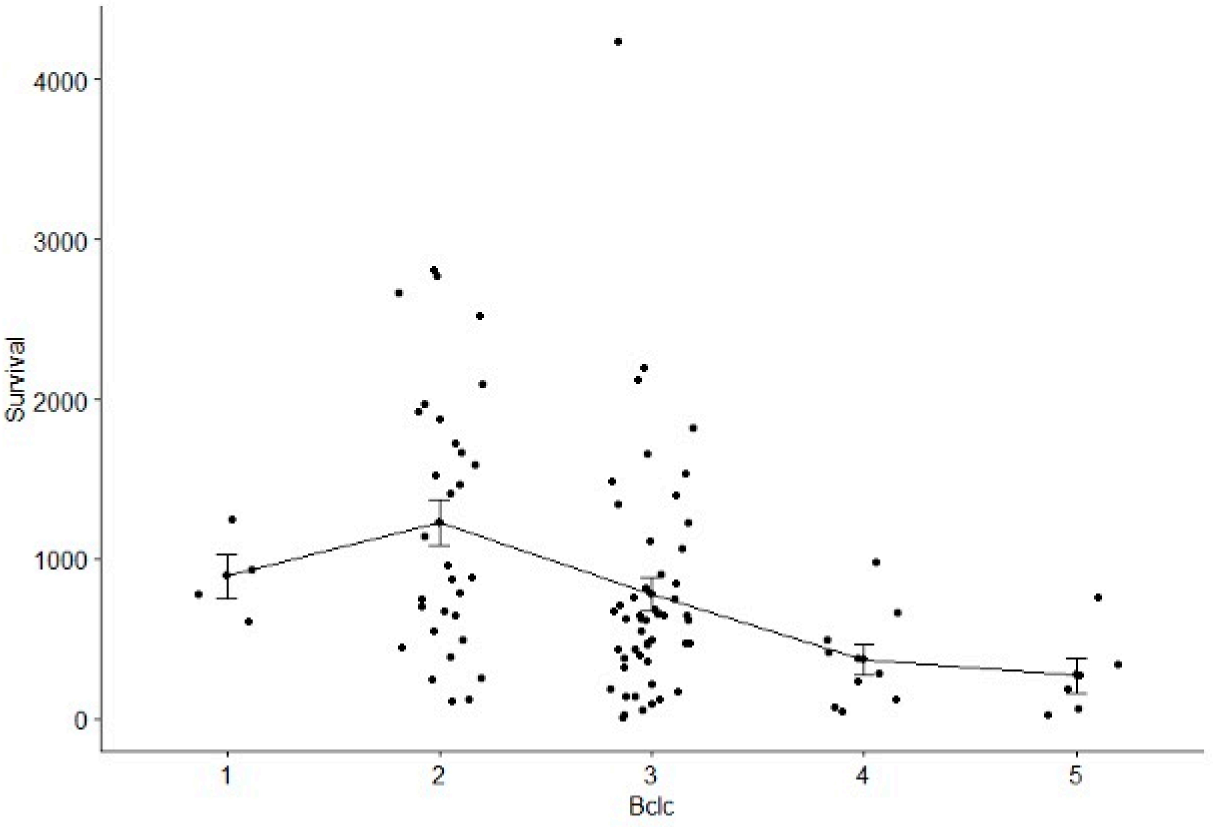

Figure S2. Scatter plot of patient survival in days according to BCLC subgroup.

Table S1. Pairwise Mann-Whitney tests.

	BCLC 0	BCLC A	BCLC B	BCLC
BCLC A	0.635	-	-	-
BCLC B	0.301	0.018	-	-
BCLC C	0.048	0.007	0.052	-
BCLC D	0.048	0.01	0.048	0.475
